# Editorial on psychoanalytical neuroscience: exploring psychoanalytic concepts with neuroscientific methods

**DOI:** 10.3389/fnhum.2014.00674

**Published:** 2014-08-28

**Authors:** Nikolai Axmacher, Henrik Kessler, Gerd T. Waldhauser

**Affiliations:** ^1^Department of Epileptology, University of BonnBonn, Germany; ^2^Department of Psychosomatic Medicine and Psychotherapy, LWL University Hospital, Ruhr-University BochumBochum, Germany; ^3^Department of Psychology, University of KonstanzKonstanz, Germany

**Keywords:** psychoanalysis, neuroscience, neuropsychoanalysis, psychodynamic psychotherapy, neuroimaging

The Research Topic “Psychoanalytical neuroscience: Exploring psychoanalytic concepts with neuroscientific methods” was cross-linked in two Frontiers journals, *Frontiers in Human Neuroscience* and *Frontiers in Psychoanalysis and Neuropsychoanalysis*. Thus, contributing authors could choose to submit to either of those two journals. Among the 16 finally accepted contributions, 14 were submitted to *Frontiers in Human Neuroscience* and 2 to *Frontiers in Psychoanalysis and Neuropsychoanalysis* (potentially related to the fact that during the time of submission only the former journal was listed with an impact factor; this has changed by now). These articles covered a wide range of topics, from empirical studies on basic psychoanalytic concepts (*n* = 4) to articles on the neurobiological mechanisms of psychodynamic therapy (*n* = 3) and theoretical reviews (*n* = 9).

Studies from the first group investigated the empirical basis of specific psychoanalytic concepts such as repression (using fMRI; Kehyayan et al., 2013), unconscious conflict (using EEG; Shevrin et al., 2013), dreams (using questionnaires; Mota-Rolim et al., 2013) or personality structure related to depression (using fMRI; Taubner et al., 2013). Studies from the second group adopted a broader, more clinical perspective and explored neuronal changes during psychodynamic therapy. Therapy involves various complex changes in psychical structure. Among those, articles in the Research Topic mainly reported on therapy-induced reductions of defenses (Buchheim et al., 2013; de Greck et al., 2013) and changes in dream content (Fischmann et al., 2013). These two approaches—studies on psychoanalytic concepts and on psychotherapy effects—have already led to interesting convergences: For example, de Greck et al. (2013) described a normalization of initially reduced activity of the medial temporal lobe after psychodynamic therapy in patients with somatoform disorders; the same region was found to be inhibited in an experimental model of repression (Schmeing et al., 2013). We believe that in the future, it will remain necessary to combine these two approaches in order to link psychoanalysis—which is before all a very specific clinical intervention—with experimental neuroscientific research.

In addition to empirical work, the Research Topic includes various theoretical articles. One repeating theme among them was the relevance of using individualized stimuli to allow for a neuroscientific investigation of subjective “meaning” that is central to psychodynamic approaches (Boeker et al., 2013; Kessler et al., 2013; Cusumano and Raz, 2014). Other topics involved the investigation of unconscious memory processes (Ruby, 2013) and the combination of psychotherapy with EEG neurofeedback (Unterrainer et al., 2013). Further theoretical accounts included a psychoanalytic framework of addiction (Johnson, 2013), a neurobiological theory of the Lacanian concept of jouissance (Bazan and Detandt, 2013), considerations of a potential relationship between microglia and the Freudian “death drive” (Kato and Kanba, 2013), and a review on the use of psychedelic drugs to examine psychoanalytic concepts (Carhart-Harris et al., 2014).

All authors were asked to elaborate on their view of the potential benefit of linking psychoanalysis and neuroscience, which was formulated by the following questions: “*First, from the neuroscientific side, why should researchers in the neurosciences address psychoanalytic ideas, and what is (or will be) the impact of this connection on current neuroscientific theories? Second, from the psychoanalytic side, why should psychoanalysts care about neuroscientific studies, and (how) can current psychoanalytical theory and practice benefit from their results?*” As expected, authors responded differently to this question. Some argued that addressing psychoanalytic concepts is beneficial to advance neuroscientific research: It may allow for an explanation of results which are otherwise difficult to interpret, and enhance the realm of processes that can be investigated using neuroscientific methods (Kehyayan et al., 2013; Ruby, 2013)—in particular, personal meaning (Boeker et al., 2013; Kessler et al., 2013; Shevrin et al., 2013; Cusumano and Raz, 2014). Others suggest psychoanalysis as a useful framework to better understand, prevent and treat psychiatric diseases such as addiction (Johnson, 2013) or depression (Taubner et al., 2013).

Other authors argued that psychoanalysis could also benefit from neuroscientific research. A relatively direct link was described by Unterrainer et al. (2013), who suggested that a combination of neurofeedback with psychodynamic psychotherapy is more beneficial than either treatment alone. On a more theoretical level, results from neuroimaging studies on psychotherapeutic treatment may allow one to disentangle the complex processes during psychotherapy, by relating the brain activation patterns to results from previous experimental studies—for example, linking them to previous research on interpersonal attachment (Buchheim et al., 2013), self-related processing (Fischmann et al., 2013), or emotional memory (de Greck et al., 2013). Although such reverse inference has been criticized due to the lack of specificity of neural activation patterns, its viability can be formally tested (Poldrack, 2011; Hutzler, 2014). Furthermore, neuroscience research may break down complex psychoanalytic concepts into biological processes that are easier to grasp: Carhart-Harris et al. (2014) proposed that application of psychedelic drugs during neuroimaging allows for an experimental investigation of primary process thinking, and Bazan and Detandt (2013) and Kato and Kanba (2013) suggested that neuroscientific findings would help to better understand complex psychoanalytic concepts such as jouissance and the death drive, respectively. The resulting integrated neuro-psychoanalytic concepts may then contribute to the development of a new metapsychology based on current neuroscientific knowledge.

We were glad to learn that our Research Topic raised considerable interest. This is not only reflected in the relatively high number of contributions; in addition, one of the authors (NA) was pleased to see it summarized in a presentation at the annual International Neuropsychoanalysis Congress in New York City by Mark Fisher entitled “*Toward a neuroscience theory of psychoanalysis: open road or dead end?*”. We believe the articles in this topic are good evidence that the emerging field of psychoanalytical neuroscience is an open road rather than a dead end: While psychoanalysis allows neuroscientific researchers to embrace the full complexity of human subjective experience and its determination by unconscious conflicts, results from the neurosciences may eventually provide psychoanalysis with a new metapsychological framework. Much work remains to be done down the road, though.

## Conflict of interest statement

The authors declare that the research was conducted in the absence of any commercial or financial relationships that could be construed as a potential conflict of interest.
